# Color and psychological functioning: a review of theoretical and empirical work

**DOI:** 10.3389/fpsyg.2015.00368

**Published:** 2015-04-02

**Authors:** Andrew J. Elliot

**Affiliations:** Department of Clinical and Social Sciences in Psychology, University of Rochester, Rochester, NYUSA

**Keywords:** color, psychological functioning, hue, lightness, chroma

## Abstract

In the past decade there has been increased interest in research on color and psychological functioning. Important advances have been made in theoretical work and empirical work, but there are also important weaknesses in both areas that must be addressed for the literature to continue to develop apace. In this article, I provide brief theoretical and empirical reviews of research in this area, in each instance beginning with a historical background and recent advancements, and proceeding to an evaluation focused on weaknesses that provide guidelines for future research. I conclude by reiterating that the literature on color and psychological functioning is at a nascent stage of development, and by recommending patience and prudence regarding conclusions about theory, findings, and real-world application.

The past decade has seen enhanced interest in research in the area of color and psychological functioning. Progress has been made on both theoretical and empirical fronts, but there are also weaknesses on both of these fronts that must be attended to for this research area to continue to make progress. In the following, I briefly review both advances and weaknesses in the literature on color and psychological functioning.

## Theoretical Work

### Background and Recent Developments

Color has fascinated scholars for millennia (Sloane, 1991; Gage, 1993). Theorizing on color and psychological functioning has been present since Goethe (1810) penned his *Theory of Colors*, in which he linked color categories (e.g., the “plus” colors of yellow, red–yellow, yellow–red) to emotional responding (e.g., warmth, excitement). Goldstein (1942) expanded on Goethe’s intuitions, positing that certain colors (e.g., red, yellow) produce systematic physiological reactions manifest in emotional experience (e.g., negative arousal), cognitive orientation (e.g., outward focus), and overt action (e.g., forceful behavior). Subsequent theorizing derived from Goldstein’s ideas has focused on wavelength, positing that longer wavelength colors feel arousing or warm, whereas shorter wavelength colors feel relaxing or cool (Nakashian, 1964; Crowley, 1993). Other conceptual statements about color and psychological functioning have focused on general associations that people have to colors and their corresponding influence on downstream affect, cognition, and behavior (e.g., black is associated with aggression and elicits aggressive behavior; Frank and Gilovich, 1988; Soldat et al., 1997). Finally, much writing on color and psychological functioning has been completely atheoretical, focused exclusively on finding answers to applied questions (e.g., “What wall color facilitates worker alertness and productivity?”). The aforementioned theories and conceptual statements continue to motivate research on color and psychological functioning. However, several other promising theoretical frameworks have also emerged in the past decade, and I review these frameworks in the following.

Hill and Barton (2005) noted that in many non-human animals, including primate species, dominance in aggressive encounters (i.e., superior physical condition) is signaled by the bright red of oxygenated blood visible on highly vascularized bare skin. Artificial red (e.g., on leg bands) has likewise been shown to signal dominance in non-human animals, mimicking the natural physiological process (Cuthill et al., 1997). In humans in aggressive encounters, a testosterone surge produces visible reddening on the face and fear leads to pallor (Drummond and Quay, 2001; Levenson, 2003). Hill and Barton (2005) posited that the parallel between humans and non-humans present at the physiological level may extend to artificial stimuli, such that wearing red in sport contests may convey dominance and lead to a competitive advantage.

Other theorists have also utilized a comparative approach in positing links between skin coloration and the evaluation of conspecifics. Changizi et al. (2006) and Changizi (2009) contend that trichromatic vision evolved to enable primates, including humans, to detect subtle changes in blood flow beneath the skin that carry important information about the emotional state of the conspecific. Increased red can convey anger, embarrassment, or sexual arousal, whereas increased bluish or greenish tint can convey illness or poor physiological condition. Thus, visual sensitivity to these color modulations facilitates various forms of social interaction. In similar fashion, Stephen et al. (2009) and Stephen and McKeegan (2010) propose that perceivers use information about skin coloration (perhaps particularly from the face, Tan and Stephen, 2012) to make inferences about the attractiveness, health, and dominance of conspecifics. Redness (from blood oxygenization) and yellowness (from carotenoids) are both seen as facilitating positive judgments. Fink et al. (2006) and Fink and Matts (2007) posit that the homogeneity of skin coloration is an important factor in evaluating the age, attractiveness, and health of faces.

Elliot and Maier (2012) have proposed color-in-context theory, which draws on social learning, as well as biology. Some responses to color stimuli are presumed to be solely due to the repeated pairing of color and particular concepts, messages, and experiences. Others, however, are presumed to represent a biologically engrained predisposition that is reinforced and shaped by social learning. Through this social learning, color associations can be extended beyond natural bodily processes (e.g., blood flow modulations) to objects in close proximity to the body (e.g., clothes, accessories). Thus, for example, red may not only increase attractiveness evaluations when viewed on the face, but also when viewed on a shirt or dress. As implied by the name of the theory, the physical and psychological context in which color is perceived is thought to influence its meaning and, accordingly, responses to it. Thus, blue on a ribbon is positive (indicating first place), but blue on a piece of meat is negative (indicating rotten), and a red shirt may enhance the attractiveness of a potential mate (red = sex/romance), but not of a person evaluating one’s competence (red = failure/danger).

Meier and Robinson (2005) and Meier (in press) have posited a conceptual metaphor theory of color. From this perspective, people talk and think about abstract concepts in concrete terms grounded in perceptual experience (i.e., they use metaphors) to help them understand and navigate their social world (Lakoff and Johnson, 1999). Thus, anger entails reddening of the face, so anger is metaphorically described as “seeing red,” and positive emotions and experiences are often depicted in terms of lightness (rather than darkness), so lightness is metaphorically linked to good (“seeing the light”) rather than bad (“in the dark”). These metaphoric associations are presumed to have implications for important outcomes such as morality judgments (e.g., white things are viewed as pure) and stereotyping (e.g., dark faces are viewed more negatively).

For many years it has been known that light directly influences physiology and increases arousal (see Cajochen, 2007, for a review), but recently theorists have posited that such effects are wavelength dependent. Blue light, in particular, is posited to activate the melanopsin photoreceptor system which, in turn, activates the brain structures involved in sub-cortical arousal and higher-order attentional processing (Cajochen et al., 2005; Lockley et al., 2006). As such, exposure to blue light is expected to facilitate alertness and enhance performance on tasks requiring sustained attention.

### Evaluation and Recommendations

Drawing on recent theorizing in evolutionary psychology, emotion science, retinal physiology, person perception, and social cognition, the aforementioned conceptualizations represent important advances to the literature on color and psychological functioning. Nevertheless, theory in this area remains at a nascent level of development, and the following weaknesses may be identified.

First, the focus of theoretical work in this area is either extremely specific or extremely general. A precise conceptual proposition such as red signals dominance and leads to competitive advantage in sports (Hill and Barton, 2005) is valuable in that it can be directly translated into a clear, testable hypothesis; however, it is not clear how this specific hypothesis connects to a broader understanding of color–performance relations in achievement settings more generally. On the other end of the spectrum, a general conceptualization such as color-in-context theory (Elliot and Maier, 2012) is valuable in that it offers several widely applicable premises; however, these premises are only vaguely suggestive of precise hypotheses in specific contexts. What is needed are mid-level theoretical frameworks that comprehensively, yet precisely explain and predict links between color and psychological functioning in specific contexts (for emerging developments, see Pazda and Greitemeyer, in press; Spence, in press; Stephen and Perrett, in press).

Second, the extant theoretical work is limited in scope in terms of range of hues, range of color properties, and direction of influence. Most theorizing has focused on one hue, red, which is understandable given its prominence in nature, on the body, and in society (Changizi, 2009; Elliot and Maier, 2014); however, other hues also carry important associations that undoubtedly have downstream effects (e.g., blue: Labrecque and Milne, 2012; green: Akers et al., 2012). Color has three basic properties: hue, lightness, and chroma (Fairchild, 2013). Variation in any or all of these properties could influence downstream affect, cognition, or behavior, yet only hue is considered in most theorizing (most likely because experientially, it is the most salient color property). Lightness and chroma also undoubtedly have implications for psychological functioning (e.g., lightness: Kareklas et al., 2014; chroma: Lee et al., 2013); lightness has received some attention within conceptual metaphor theory (Meier, in press; see also Prado-León and Rosales-Cinco, 2011), but chroma has been almost entirely overlooked, as has the issue of combinations of hue, lightness, and chroma. Finally, most theorizing has focused on color as an independent variable rather than a dependent variable; however, it is also likely that many situational and intrapersonal factors influence color perception (e.g., situational: Bubl et al., 2009; intrapersonal: Fetterman et al., 2015).

Third, theorizing to date has focused primarily on main effects, with only a modicum of attention allocated to the important issue of moderation. As research literatures develop and mature, they progress from a sole focus on “is” questions (“Does X influence Y?”) to additionally considering “when” questions (“Under what conditions does X influence Y and under what conditions does X *not* influence Y?”). These “second generation” questions (Zanna and Fazio, 1982, p. 283) can seem less exciting and even deflating in that they posit boundary conditions that constrain the generalizability of an effect. Nevertheless, this step is invaluable in that it adds conceptual precision and clarity, and begins to address the issue of real-world applicability. All color effects undoubtedly depend on certain conditions – culture, gender, age, type of task, variant of color, etc. – and acquiring an understanding of these conditions will represent an important marker of maturity for this literature (for movement in this direction, see Schwarz and Singer, 2013; Tracy and Beall, 2014; Bertrams et al., 2015; Buechner et al., in press; Young, in press). Another, more succinct, way to state this third weakness is that theorizing in this area needs to take context, in all its forms, more seriously.

## Empirical Work

### Background and Recent Developments

Empirical work on color and psychological functioning dates back to the late 19th century (Féré, 1887; see Pressey, 1921, for a review). A consistent feature of this work, from its inception to the past decade, is that it has been fraught with major methodological problems that have precluded rigorous testing and clear interpretation (O’Connor, 2011). One problem has been a failure to attend to rudimentary scientific procedures such as experimenter blindness to condition, identifying, and excluding color deficient participants, and standardizing the duration of color presentation or exposure. Another problem has been a failure to specify and control for color at the spectral level in manipulations. Without such specification, it is impossible to know what precise combination of color properties was investigated, and without such control, the confounding of focal and non-focal color properties is inevitable (Whitfield and Wiltshire, 1990; Valdez and Mehrabian, 1994). Yet another problem has been the use of underpowered samples. This problem, shared across scientific disciplines (Maxwell, 2004), can lead to Type I errors, Type II errors, and inflated effect sizes (Fraley and Vazire, 2014; Murayama et al., 2014). Together, these methodological problems have greatly hampered progress in this area.

Although some of the aforementioned problems remain (see “Evaluation and Recommendations” below), others have been rectified in recent work. This, coupled with advances in theory development, has led to a surge in empirical activity. In the following, I review the diverse areas in which color work has been conducted in the past decade, and the findings that have emerged. Space considerations require me to constrain this review to a brief mention of central findings within each area. I focus on findings with humans (for reviews of research with non-human animals, see Higham and Winters, in press; Setchell, in press) that have been obtained in multiple (at least five) independent labs. **Table 1** provides a summary, as well as representative examples and specific references.

**Table 1:** Research on color and psychological functioning.

Area of research	Central finding	Example	References
Color and Selective Attention	Red stimuli have been shown to receive an attentional advantage	Participants’ visual search times were faster for desaturated red (relative to several other colored) targets	Lindsay et al., 2010; Tchernikov and Fallah, 2010; Buechner et al., 2014; Pomerleau et al., 2014; Sokolik et al., 2014 (cf. Becker et al., 2014; Folk, in press)
Color and Alertness	Blue light has been shown to increase subjective alertness and performance on attention-based tasks	Participants exposed to blue (relative to yellow) illumination reported greater mental alertness	Lockley et al., 2006; Lehrl et al., 2007; Viola et al., 2008; Cajochen et al., 2011; Taillard et al., 2012 (cf. Vandewalle et al., 2007; Sahin and Figuerio, 2013)
Color and Athletic Performance	Wearing red has been shown to enhance performance and perceived performance in sport competitions and tasks	Tae kwon do competitors wearing red outperformed those wearing blue	Hill and Barton, 2005; Hagemann et al., 2008; Ilie et al., 2008; Greenlees et al., 2013; Sorokowski et al., 2014 (cf. Caldwell and Burger, 2011; Garcia-Rubio et al., 2011)
Color and Intellectual Performance	Viewing red prior to a challenging cognitive task has been shown to undermine performance	Participants who viewed red (relative to green or gray) on an intelligence test cover performed worse on the test	Elliot et al., 2007; Gnambs et al., 2010; Zhang and Han, 2014; Shi et al., 2015; Thorstenson, in press (cf. Yamazaki, 2010; Smajic et al., 2014)
Color and Aggressiveness/Dominance Evaluation	Viewing red on self or other has been shown to increase appraisals of aggressiveness/dominance	Participants rated males wearing red (relative to other chromatic colors) as more dominant	Greenlees et al., 2008; Little and Hill, 2007; Feltman and Elliot, 2011; Stephen et al., 2012a; Aiken and Pascal, 2013 (cf. Sorokowski and Szmajke, 2007; Furley et al., 2012)
Color and Avoidance Motivation	Viewing red in achievement contexts has been shown to increase caution and avoidance	Participants who viewed red (relative to green or gray) prior to an ostensible intelligence test evidenced greater right (versus left) frontal cortical activation	Elliot et al., 2007; Mehta and Zhu, 2009; Rutchick et al., 2010; Tanaka and Tokuno, 2011; Ten Velden et al., 2012 (cf. Elwood and Bode, 2014; Steele, 2014)
Color and Attraction	Viewing red on or near a female has been shown to increase attraction in heterosexual males	Heterosexual males rated females wearing red (relative to other chromatic colors) as more attractive	Elliot and Niesta, 2008; Roberts et al., 2010; Stephen and McKeegan, 2010; Guéguen and Jacob, 2014; Lin, 2014 (cf. Lynn et al., in press; Stephen et al., 2012b)
Color and Store/Company Evaluation	Blue stores/logos have been shown to increase quality and trustworthiness appraisals	Participants rated websites featuring blue (relative to green) as more trustworthy	Yüksel, 2009; Lee and Rao, 2010; Alberts and van der Geest, 2011; Labrecque and Milne, 2012; Ridgway and Myers, 2014 (cf. Barli et al., 2006; Chebat and Morrin, 2007)
Color and Eating/Drinking	Red has been shown to influence food and beverage perception and consumption	Participants ate less chocolate chips from a red (relative to blue or white) plate	Ross et al., 2008; Genschow et al., 2012; Guéguen, 2012; Bruno et al., 2013; Spence et al., 2014 (cf. Piqueras-Fiszman et al., 2012; Van Ittersum and Wansink, 2012)

*The review of findings was restricted to those that have been supported by a minimum of five independent laboratories. The references are to representative articles within each area of research; articles with supportive findings area listed first, followed by articles with non-supportive findings (indicated by cf.).*

In research on color and selective attention, red stimuli have been shown to receive an attentional advantage (see Folk, in press, for a review). Research on color and alertness has shown that blue light increases subjective alertness and performance on attention-based tasks (see Chellappa et al., 2011, for a review). Studies on color and athletic performance have linked wearing red to better performance and perceived performance in sport competitions and tasks (see Maier et al., in press, for a review). In research on color and intellectual performance, viewing red prior to a challenging cognitive task has been shown to undermine performance (see Shi et al., 2015, for a review). Research focused on color and aggressiveness/dominance evaluation has shown that viewing red on self or other increases appraisals of aggressiveness and dominance (see Krenn, 2014, for a review). Empirical work on color and avoidance motivation has linked viewing red in achievement contexts to increased caution and avoidance (see Elliot and Maier, 2014, for a review). In research on color and attraction, viewing red on or near a female has been shown to enhance attraction in heterosexual males (see Pazda and Greitemeyer, in press, for a review). Research on color and store/company evaluation has shown that blue on stores/logos increases quality and trustworthiness appraisals (see Labrecque and Milne, 2012, for a review). Finally, empirical work on color and eating/drinking has shown that red influences food and beverage perception and consumption (see Spence, in press, for a review).

### Evaluation and Recommendations

The aforementioned findings represent important contributions to the literature on color and psychological functioning, and highlight the multidisciplinary nature of research in this area. Nevertheless, much like the extant theoretical work, the extant empirical work remains at a nascent level of development, due, in part, to the following weaknesses.

First, although in some research in this area color properties are controlled for at the spectral level, in most research it (still) is not. Color control is typically done improperly at the device (rather than the spectral) level, is impossible to implement (e.g., in web-based platform studies), or is ignored altogether. Color control is admittedly difficult, as it requires technical equipment for color assessment and presentation, as well as the expertise to use it. Nevertheless, careful color control is essential if systematic scientific work is to be conducted in this area. Findings from uncontrolled research can be informative in initial explorations of color hypotheses, but such work is inherently fraught with interpretational ambiguity (Whitfield and Wiltshire, 1990; Elliot and Maier, 2014) that must be subsequently addressed.

Second, color perception is not only a function of lightness, chroma, and hue, but also of factors such as viewing distance and angle, amount and type of ambient light, and presence of other colors in the immediate background and general environmental surround (Hunt and Pointer, 2011; Brainard and Radonjić, 2014; Fairchild, 2015). In basic color science research (e.g., on color physics, color physiology, color appearance modeling, etcetera; see Gegenfurtner and Ennis, in press; Johnson, in press; Stockman and Brainard, in press), these factors are carefully specified and controlled for in order to establish standardized participant viewing conditions. These factors have been largely ignored and allowed to vary in research on color and psychological functioning, with unknown consequences. An important next step for research in this area is to move to incorporate these more rigorous standardization procedures widely utilized by basic color scientists. With regard to both this and the aforementioned weakness, it should be acknowledged that exact and complete control is not actually possible in color research, given the multitude of factors that influence color perception (Committee on Colorimetry of the Optical Society of America, 1953) and our current level of knowledge about and ability to control them (Fairchild, 2015). As such, the standard that must be embraced and used as a guideline in this work is to control color properties and viewing conditions to the extent possible given current technology, and to keep up with advances in the field that will increasingly afford more precise and efficient color management.

Third, although in some research in this area, large, fully powered samples are used, much of the research remains underpowered. This is a problem in general, but it is particularly a problem when the initial demonstration of an effect is underpowered (e.g., Elliot and Niesta, 2008), because initial work is often used as a guide for determining sample size in subsequent work (both heuristically and via power analysis). Underpowered samples commonly produce overestimated effect size estimates (Ioannidis, 2008), and basing subsequent sample sizes on such estimates simply perpetuates the problem. Small sample sizes can also lead researchers to prematurely conclude that a hypothesis is disconfirmed, overlooking a potentially important advance (Murayama et al., 2014). Findings from small sampled studies should be considered preliminary; running large sampled studies with carefully controlled color stimuli is essential if a robust scientific literature is to be developed. Furthermore, as the “evidentiary value movement” (Finkel et al., 2015) makes inroads in the empirical sciences, color scientists would do well to be at the leading edge of implementing such rigorous practices as publically archiving research materials and data, designating exploratory from confirmatory analyses, supplementing or even replacing significant testing with “new statistics” (Cumming, 2014), and even preregistering research protocols and analyses (see Finkel et al., 2015, for an overview).

## Conclusion

In both reviewing advances in and identifying weaknesses of the literature on color and psychological functioning, it is important to bear in mind that the existing theoretical and empirical work is at an early stage of development. It is premature to offer any bold theoretical statements, definitive empirical pronouncements, or impassioned calls for application; rather, it is best to be patient and to humbly acknowledge that color psychology is a uniquely complex area of inquiry (Kuehni, 2012; Fairchild, 2013) that is only beginning to come into its own. Findings from color research can be provocative and media friendly, and the public (and the field as well) can be tempted to reach conclusions before the science is fully in place. There is considerable promise in research on color and psychological functioning, but considerably more theoretical and empirical work needs to be done before the full extent of this promise can be discerned and, hopefully, fulfilled.

## Conflict of Interest Statement

The author declares that the research was conducted in the absence of any commercial or financial relationships that could be construed as a potential conflict of interest.
